# Paracentesis Pericardii

**Published:** 1892-07-30

**Authors:** 


### The Practitioner's Mirror and Retrospect.

[*The Editor will be glad to receive offers of co-operation and contributions from members of the profession. All letters should be addressed to* The Editor, The Lodge, Porchester Square, London, W.]

### MEDICINE.

### PARACENTESIS PERICARDII.

We recently referred to cases in which incision and drainage of the pericardium had been undertaken successfully for purulent pericarditis, and the good results which attended this bold innovation in surgical practice demonstrated that the general and fundamental principles of surgery are applicable to the pericardium. Of course such a procedure will only be undertaken when the urgency of symptoms leave no doubt that nothing short of the opening of the pericardium will avail to save the life of the patient, and the profession has been so deeply imbued with the idea that the pericardium is practically outside the domain of surgery, that, even in such desperate circumstances as we have pre-supposed, it is to be feared that the patient will often be left to die without even attempted relief. Even the milder operative procedure of paracentesis pericardii is so rarely undertaken that we cannot avoid the conclusion that we are still much too averse to resorting to it in cases of large pericardial effusion with greatly embarrassed heart's action.

Dr. Churton, of Leeds, has recently reported a case of hœmorrhagic pericarditis,* in which frequent aspirations were followed by recovery. A man, aged 46, was admitted into Leeds Infirmary on July 24th, 1890, with dyspnœa and pain in the chest. There were signs of fluid in the right pleura and in the pericardium. The chest wall was distinctly œdematous; no œdema elsewhere. Patient had taken very little solid food for fifteen years, living chiefly on beer and whiskey. Twenty-nine ounces of clear fluid were withdrawn from the right pleura without giving any relief, and no less than five subsequent attempts to substitute tapping of the pleura for aspiration of the pericardium always similarly failed to effect any improvement. The pericardium was then explored, (1) in the left fifth space, just external to the mammary line; (2) half an inch nearer the median line; (3) in the right fifth space, an inch from the edge of the sternum. Deeply blood-stained fluid was found at each point. The pericardium was then aspirated by Mr. Mayo Robson in the left fifth space in the nipple line, eight ounces being obtained. The fluid deposited one-twelfth blood cells. The following day Mr. Moynam aspirated drawing off three ounces, but the pulse fell from 128 to 90. The improvement was as transitory as before; a further aspiration removed another two ounces of fluid. The author, like many others, had frequently observed in the post-mortem room that fluid in large quantity, and free in the pericardial cavity was collected in three chief pools — one in the right lower angle, another in the left, the third at the upper part of the cavity; and, as it was evident that a needle passed too deeply into either of the lateral pools would merely pass harmlessly through the back of the pericardium into the lung, and as measurements showed that this point would, in a distended pericardium, be about two inches and a half from the surface, he, on August 8th, passed an aspirating needle to a depth of two inches in the left fifth space, in the nipple line; twenty-two ounces of similar fluid was thus obtained. The chest was raised before the aspiration in order to bring more of the fluid to the lower part of the pericardium. The relief though great was again of short duration, as the patient had almost invincible anorexia. Five subsequent aspirations were made at intervals of a day or two. He was described by the house surgeon as "rambling, moaning, pallid, and wan, seems unconscious, but is easily roused for a few moments; excreta are passed in the bed; radial pulse 100, scarcely perceptible; respiration, 36." A few days before the last aspiration but one, dry cupping over the præcordium was ordered daily, and this apparently had a good effect; the fluid soon became evidently less; there seemed to be some present, however, even four months afterwards. The patient was in bed six months and in the hospital eight months, after which he was at a convalescent home for six weeks.

* *Lancet*, Nov. 21st, 1891.

Another case recently reported by Shattock * was that of a young woman, aged 24, who was suffering from rheumatic symptoms and pericarditis. The pericardial effusion rapidly increased, so that on the eighth day the area of cardiac dulness extended from well outside the left nipple nearly to the right nipple and up to her second rib. Aspiration was decided upon, so a trocar was introduced into the fifth left interspace three inches from the median line; about one ounce of bloody serous fluid was withdrawn. As no more could be obtained from this situation, though the cannula was freely moveable, another puncture was made in the fourth right space, two and a-half inches from the middle line, but no fluid was obtained. An ice bag was applied to the heart, and next day pericardial friction was heard. Two days later, as she was worse, another puncture was made on the left side, but only an ounce of blood-stained serum was withdrawn; and two days later another puncture on this side, but further out, failed to remove any fluid. After this the patient did well and eventually recovered.

Now these two cases, which we have referred to in some detail because they illustrate the difficulties that may have to be encountered, cannot fail to be instuctive. In both cases a good recovery was eventually obtained, but it is clear that we must not expect that a single puncture will effect relief. It is very evident that to lay down definite rules as to the seat of puncture in paracentesis pericardii would be very misleading. Nevertheless, puncture at a spot about two to two and a half inches to the left from the edge of the sternum, in the fifth or sixth interspaces, will prove the most likely place where paracentesis will prove successful in relieving pericardial distension. It is well to be careful to abstain from pushing the needle any deeper than is necessary, and having entered the pericardial sac the needle must be pushed obliquely upwards, and steadied so as to avoid the risk of wounding the heart. An interesting case has been reported by Dziembouski, in which a sewing needle had entered the pericardium of a boy and set up a loud pericardial friction sound, audible nearly a foot away from the patient's chest. An incision down to the pericardium exposed the broken end of the needle, which was removed, being followed by a rapid and perfect recovery of the patient.

* *Boston Med. and Surg. Journal*, Nov. 5th, 1891; *Brit. Med. Journal* Jan. 30th, 1892.

